# New cell sources for CAR-based immunotherapy

**DOI:** 10.1186/s40364-023-00482-9

**Published:** 2023-05-06

**Authors:** Marzieh Mazinani, Fatemeh Rahbarizadeh

**Affiliations:** 1 Department of Medical Biotechnology, Faculty of Medical Sciences, Tarbiat Modares University, P.O. Box 14115-111, Tehran, Iran; 2 Research and Development Center of Biotechnology, Tarbiat Modares University, Tehran, Iran

**Keywords:** Chimeric antigen receptors, Regulatory T cells, γδT cells, Mucosal-associated invariant T cells, Natural killer T cells, Natural killer cells, Macrophages, Neutrophils, Hematopoietic stem/progenitor cells, Induced pluripotent stem cells

## Abstract

Chimeric antigen receptor (CAR) T cell therapy, in which a patient’s own T lymphocytes are engineered to recognize and kill cancer cells, has achieved striking success in some hematological malignancies in preclinical and clinical trials, resulting in six FDA-approved CAR-T products currently available in the market. Despite impressive clinical outcomes, concerns about treatment failure associated with low efficacy or high cytotoxicity of CAR-T cells remain. While the main focus has been on improving CAR-T cells, exploring alternative cellular sources for CAR generation has garnered growing interest. In the current review, we comprehensively evaluated other cell sources rather than conventional T cells for CAR generation.

## Introduction

Chimeric antigen receptor (CAR) T cell therapy, in which a patient’s T lymphocytes are genetically modified to recognize and eradicate tumor cells independent of the major histocompatibility complex (MHC), has achieved remarkable success in some hematological malignancies, leading to the regulatory approval of six CAR-T products, including Tisagenlecleucel (Kymriah®), Axicabtagene ciloleucel (Yescarta®), Brexucabtagene autoleucel (Tecartus®), Lisocabtagene maraleucel (Breyanzi®), Idecabtagene vicleucel (ABECMA®), and Ciltacabtagene autoleucel (CARVYKTI®), that are currently available in the market [1–6]. CARs are synthetic immune receptors that benefit from both humoral and adaptive immunity through their unique structure at which an extracellular antigen recognition domain, typically a single-chain variable fragment (scFv) derived from a monoclonal antibody, is linked to an intracellular signaling domain of T cells. To date, the most common cell source for CAR generation used and infused into patients is conventional Τ cells derived directly from cancer patients. Despite promising results of CAR-T cell therapy reported from numerous phase I/II clinical trials conducted at single or multicentre institutions [7–9], some issues associated with the cell type described below have prompted researchers to look for alternatives [10]. The first issue is the high financial burden placed on the patient and healthcare systems due to the high cost of manufacturing a single product from a single patient using their own T-cells [11]. In 2020, the total cost of a single-dose administration of CAR-T cells in hematological malignancy, which includes lymphodepletion, the acquisition and infusion of CAR-T cells, and management of acute adverse events, is estimated to be around USD 454,611 in an academic hospital inpatient setting [12]. The second issue is the manufacturing time. The manufacturing process, which includes T-cell activation, viral transduction, and at least six days of ex vivo expansion, roughly takes two weeks [11, 13]. This two-week is a vulnerable time for patients, particularly those experiencing rapidly-progressing diseases in environments with limited resources [14]. The next issue is patient access to the product that is currently manufactured in a centralized mode. Although Ghassemi et al. developed a method to generate highly functional CAR-T cells within 24 h that can significantly reduce the cost and timeframe associated with CAR-T cell manufacturing and decentralize it to local hospital laboratories [15], this highly personalized therapy may still suffer from poor quality and low efficacy of CAR-T cells produced from heavily pre-treated patients [16]. Many efforts have been made to generate allogeneic CAR-T products using high-quality T cells from healthy donors. Allogeneic CAR-T cells could offer many advantages over autologous (patient-derived) cells, including the potential to be cost-effective, readily available, and provide a higher quality product; however, the allogeneic cell transplantation-related issues, such as GvHD and host immune system rejection must be addressed [14, 17]. Studies showed that an inverse correlation exists between the T cell differentiation stage and alloreactivity. Memory T cells are less likely to cause GvHD in the HLA-mismatched setting than naïve T cells. For example, CAR-T cells generated from CD45RA-negative memory T cells, such as central memory (CM) or effector memory (EM) T cells, display improved effector function and decreased risk of GvHD in vitro and in vivo [18–20]. Therefore, when generating allogeneic CAR-T cells, using memory T cell subsets as the initial cell source may confer a reduced risk of GvHD. The knockout of β-2 microglobulin (B2M), a component of HLA class I molecules expressed on T cells, may reduce the immunogenicity of infused CAR-T cells. Ren et al. reported that B2M gene-disrupted CAR-T cells had reduced alloreactivity in vivo [21]. Kagoya et al. also showed that in addition to B2M knockout, eliminating CIITA, the HLA class II transactivator, improved CAR-T cell persistence in vitro [22]. Knocking out the T cell receptor (TCR) in the infused cells may also reduce the risk of GvHD [23]. Using targeted nucleases, including meganucleases, zinc-finger nucleases (ZFNs), transcription activator-like effector nucleases (TALEN), and CRISPR/Cas9 system, TCR gene disruption in human T cells is within reach [24]. Eyquem et al. developed an elegant CAR-knock-in and TCR-knockout strategy by inserting a CD19 CAR construct into the first exon of the constant chain of the TCRα gene (TRAC). This dual strategy not only averts tonic CAR signaling by optimizing CAR internalization and re-expression kinetics but also minimizes the risk of GvHD by diminishing the expression of αβTCRs on the T cell surface [25]. Allogeneic, TCR-disrupted CAR-T cells have shown feasibility in two clinical trials with relapsed B-cell leukemia, demonstrating a 67% CR (complete remission) and a 6-month PFS (progression-free-survival) of 27% [26].

Lymphodepletion chemotherapy is usually required before CAR-T cell infusion to minimize the possibility of host immune system rejection of CAR-T cells, thus prolonging their persistence [27]. Recently, Mo et al. engineered an alloimmune defense receptor (ADR) that selectively targets 4-1BB, a cell surface receptor temporarily upregulated by activated alloreactive lymphocytes. They observed that T cells co-expressing CAR and ADR resisted host cellular rejection and retained potent anti-tumor activity in vitro and in vivo [28]. Besides GvHD and the possibility of the host immune system rejection, another challenge with allogenic CAR-T cells is the risk of alloimmunization, where the recipient develops donor-specific anti-HLA antibodies (DSAs) due to donor/ recipient HLA incompatibility [29, 30]. Thus to benefit from allogenic CAR-T cells, the above-stated challenges should be managed [29].

Allogeneic CAR-T cells may address concerns related to the quality of CAR-T products, but some other issues associated with the cell type still remain. While patients undergoing CAR-T cell therapies may experience a temporary or even durable complete remission of their cancer after receiving lymphodepletion chemotherapy followed by CAR-T cell infusion, they may develop unique acute toxicities, such as CRS (cytokine release syndrome) and neurotoxicity that may require early recognition by the oncologic team with timely involvement of critical care teams or further treatment in an intensive care unit (ICU) to avoid their progression into other life-threatening complications [31]. Also, unlike patients with hematological malignancies that benefit the most from CAR-T cell therapies [7–9, 32, 33], these cell products have not shown satisfying response rates against solid tumors. Moreover, CAR-T cells targeting a single specific tumor antigen may provide a prime opportunity for tumor immune evasion through antigen loss, a particular issue in anti-CD19 CAR-T therapy [34]. Development of exhaustion and limited persistence of CAR-T cells is also a big challenge that may result in an early relapse [35]. While the main focus has been on improving CAR-T cells, exploring alternative cellular sources for CAR generation has garnered growing interest. Using cells that naturally penetrate deep tumors better, eliminate them through additional mechanisms, better establish and maintain an anti-tumor microenvironment, cause minimal side effects or alloreactivity, and are less prone to antigen escape or intrinsic cytotoxic resistance, may overcome some deficiencies of CAR-T cells. Utilizing other cell types may also reduce the massive manufacturing cost and the possibility of manufacturing failures in some patients [11]. In this review, we comprehensively evaluate other cell sources rather than conventional T cells for CAR generation that include regulatory T (Treg) cells, γδT cells, Mucosal-associated invariant T cells (MAIT), natural killer T (NKT) cells, natural killer (NK) cells, macrophages, neutrophils, hematopoietic stem/progenitor cells (HSPCs), and induced pluripotent stem cells (iPSCs). The properties of cell sources mentioned in this study are summarized in Table 1.

**Table 1:** Properties of cell sources mentioned in this study

	**Tregs**	**γδT cells**	**MAITs**	**iNKT cells**	**NK cells **	**Monocyte/Macrophages **	**Neutrophils **	**HSPCs**
Phenotype	CD3 + CD4 + CD25 + FOXP3 + CD127^low^	CD3 + γδTCR	CD3 + Vα7.2 + CD161 +	CD3 + CD56 +	CD3-CD56 +	CD11b + CD14 + CD15 + CD16 +	CD11b + CD16 + CD66b +	CD34 +
Frequency in peripheral blood	1–4% of PBMCs	0.5–5% of circulating T cells	up to 10% of circulating T cells	< 1% of circulating T cells	10–15%	6% of circulating leukocytes	50–70% of circulating leukocytes	0.01–0.1%
Source	PB, UCB, Thymuse	PB	PB, HSPCs, iPSCs	PB	PB, UCB, BM, cell lines, hESCs, HSPCs, iPSCs	PB, HSPCs, iPSCs	PB, HSPCs, iPSCs	PB, UCB, BM
MHC	Restricted	Non-restricted	Non-restricted	Non-restricted	NA	NA	NA	HSPCs will produce granulocytes and monocytes within 1–2 weeks, followed by the production of NK cells in a few months and T-lymphocytes for some time. Depending on the cell produced, phenotypic and functional properties may differ
Receptors	αβTCR, TLR	γδTCR, FcRs, NKR	αβTCR, NKR	semi-invariant αβTCR, NKRs	NKRs	FcRs, TLR	FcRs, TLR	
Perforin/granzymes	Yes	Yes	Yes	Yes	Yes	No	No	
Apoptosis-inducing ligands (TRAIL, FasL, …)	Yes	Yes	Yes	Yes	Yes	No	Yes	
ADCC	No	Yes	No	No	Yes	Yes	Yes	
Pro-inflammatory cytokines (IFN-γ, TNF, GM-CSF, …)	No	Yes	Yes	Yes	Yes	Yes	Yes	
Phagocytosis	No	No	No	No	No	Yes	Yes	

*PB* Peripheral Blood, *UCB* Umbilical Cord Blood, *BM* Bone Marrow, *hESCs* Human Embryonic Stem Cells, *HSPCs* Hematopoietic Stem/Progenitor Cells, *iPSCs* Induced Pluripotent Stem Cells, *NKRs* NK Receptors, *TLRs* Toll-Like Receptors

### Regulatory T cells

#### Properties

Regulatory T cells (Tregs) are a subset of conventional αβT cell that plays a central role in maintaining homeostasis and preventing autoimmunity. They represent approximately 1–4% of peripheral blood mononuclear cells (PBMCs) [36]. Tregs are typically a subpopulation of CD4 + T cells characterized by high expressions of the IL-2 receptor α chain (CD25) and the forkhead box P3 (FOXP3) and the low level of the IL-7 receptor α chain (CD127). However, other regulatory immune cells with different phenotypes, such as CD4 + FOXP3-Tregs (Tr1 and Th3) or CD8 + Tregs, have also been identified [37, 38]. Conventional T cells can transiently express FOXP3; however, high levels of FOXP3 and demethylation of the Treg-specific-demethylated-region (TSDR), a conserved region within the FOXP3 gene, are distinct features of Tregs [39]. There are two major subsets of CD4 + CD25 + Tregs: [1] thymus-derived Tregs (tTregs) that arise in the thymus as a distinct lineage of CD4 + T cells and control tolerance to self-antigens, [2] peripheral-derived Tregs (pTregs) that develop in the periphery from conventional naive CD4 + Foxp3 − T cells upon antigen exposure under tolerogenic conditions, and seem to control immunity to foreign antigens [40–42]. Both subsets have similar phenotypic characteristics and comparable suppressive function but exhibit specific differences, including epigenetic modification of the FoxP3 gene and phenotype stability [43]. Tregs suppress immune responses through direct interaction with other immune cells, via CTLA-4 engagement as an example, or indirectly by producing immunosuppressive cytokines, such as IL-10, IL-35, and TGF-β. Furthermore, they can block T cell activation indirectly through interaction with APCs and preventing their maturation and expression of costimulatory molecules and cytokine secretion [44, 45].

#### Advantages

The ability of Tregs to suppress effector immune responses and sustain immunological homeostasis renders the adoptive transfer of these cells a promising strategy to combat a variety of diseases characterized by excessive immune activation [46, 47]. Given that they can be isolated, manipulated, and expanded in large numbers in vitro, human Tregs are attractive candidates for immunotherapeutic purposes, despite their rarity in circulation [48]. In human clinical trials, Treg infusions have shown to be safe and well-tolerated that could successfully prevent or attenuate autoimmune disease [49, 50] as well as allogeneic hematopoietic cell or solid organ transplant rejection [51, 52], thus reducing dependency on immunosuppressive drugs. However, utilizing large numbers of polyclonal Tregs with unknown antigen specificities may result in unwanted, widespread, non-specific immunosuppression, increasing the risk of acquiring opportunistic infections or cancer [53]. For example, viral reactivation after infusion of polyclonal Tregs has been reported [47]. Redirecting Tregs toward the desired antigen allows targeted suppression at lower effective doses [54]. Additionally, preclinical studies have shown antigen-specific Tregs are superior to their polyclonal counterparts in their migration to and persistence in the target tissue and in their execution of local immunosuppressive response [55–57]. In addition to isolation and expansion of endogenous antigen-specific Tregs, which is an ineffective method due to the scarcity of antigen-specific Tregs in the original polyclonal cells, engineering Tregs with recombinant TCRs is another strategy granting specificity in polyclonal Tregs [58]. Although Tregs engineered with TCRs recognize peptides from intracellular and surface-derived proteins with high affinity and induce a potent immune synapse formation, they are MHC-restricted, which limits their therapeutic applications [55]. Conversely, CAR technology offers an MHC-independent strategy to redirect Treg specificity toward pathogenic T cells or the affected tissue [55]. Several sources of human Treg cells have been explored, including peripheral blood, which is the most accessible and often the sole option for autologous applications, umbilical cord blood (UCB), and thymuses removed during pediatric cardiac surgery [55]. CAR-Tregs can be generated either by the transduction of polyclonal Tregs with CAR construct or by the co-transduction of T cells (CD3 + or CD4 +) with CAR construct and FoxP3 cDNA; however, the former strategy is limited by low levels of Tregs in peripheral blood and by the potential for downregulation of the Treg phenotype [59]. Furthermore, a recent study on CD19-specific CAR-Tregs generated from CD4 + CD25 + CD127^low^ Tregs found that both CD45RA + naïve and CD45RO + memory Tregs expressed high levels of Foxp3 immediately after isolation; however, after CAR transduction and long-term in vitro expansion, only the CD45RA + population maintained FOXP3 expression and suppression, proposing that naïve cells may make a better starting-population for CAR-Treg production [42, 60].

#### Clinical applications

Accumulated studies have reported the successful use of CAR-Tregs in preventing or attenuating GvHD and autoimmune diseases [59, 61–65]. Preclinical data from animal models have demonstrated that Tregs engineered to express an HLA–A2–specific CAR (anti-A2 CAR) successfully prevent GvHD and offer better protection from graft rejection than polyclonal Tregs [61, 62]. These promising results have led to two clinical trials using anti-A2 CAR-Tregs in the renal (NCT04817774) and liver (NCT05234190) transplant patients [59]. Studies have lately evaluated other target antigens, such as CD83 [66] and CD19 [60], or other Treg cells, such as CD8 + Tregs, as potential candidates for the suppression of GvHD [67]. Recently, Mohseni et al. found that anti-A2 CAR-Tregs equipped with the constitutive expression of IL-10 maintained a stable phenotype after transduction while suppressing alloresponses potently [68].

Unlike GvHD, CAR-Tregs have not often shown encouraging results in type 1 diabetes (T1D). For example, CAR-Tregs targeting insulin or HPi2, a human pancreatic endocrine marker, failed to prevent diabetes in xenograft models [69] or maintain expansion capacity due to persistent tonic signaling [70], respectively. However, Imam et al. showed that Tregs expressing a beta-cell antigen-specific (GAD65) CAR could successfully expand, home to pancreatic islets of humanized mouse models of the T1D, and control blood glucose to some extent [63]. In multiple sclerosis (MS), which is an autoimmune disease of the central nervous system (CNS) caused by autoreactive T cells recognizing myelin epitope, resulting in irreversible disability in more than 1 million people in the United States, CD4 + T cells modified with CAR targeting myelin oligodendrocyte glycoprotein (MOG) and murine FoxP3 gene have shown successful results in controlling a murine model of MS [64].

The application of CAR-Tregs can be expanded to more autoimmune diseases, such as autoimmune liver diseases (AILD), by identifying suitable target antigens [71]; however, Tregs have a unique characteristic of bystander suppression, an intrinsic property of broadly suppressing T cells with different antigen specificity, enabling the rational design to redirect Treg cells to inflamed tissue without necessarily targeting cell surface antigens [72]. By taking advantage of this property, Raffin et al. developed a CAR directed against citrullinated vimentin (CV), a posttranslational modified intermediate filament protein exclusively and abundantly present in the extracellular matrix of the synovial tissue of 50% of patients with rheumatoid arthritis (RA), aiming to restore homeostasis at the site of inflammation [65]. They observed that CV-specific CAR-Tregs expanded in the presence of synovial fluid from RA patients, suggesting that CV present in inflamed joints is sufficient to activate these CAR-Tregs [65].

Besides those mentioned above, CAR-Tregs have been used for the treatment of other diseases, such as hemophilia A [73, 74], vitiligo [75], inflammatory bowel disease [46, 76], asthma [77, 78], cardiovascular diseases [79] and senescence-associated pathologies [80]. They have also been employed in gene therapy to maintain transgene expression. Arjomandnejad et al. used a third-generation CAR-Treg specific for adeno-associated virus (AAV) capsid to suppress host T-cell responses against AAV capsid, a phenomenon widely observed in those clinical trials that use AAV as gene delivery tools [81]. They found that anti-AAV CAR-Tregs suppress effector T-cell proliferation and cytotoxicity in-vitro. In mouse models, anti-AAV CAR-Tregs mediated continued transgene expression, produced immunosuppressive cytokines, and decreased tissue inflammation [81]. Recently, a phase I/II clinical trial (NCT05114837) has been evaluating the safety, tolerability, and potential anti-tumor efficacy of allogeneic anti-CD19 CAR-Treg in adult patients with relapsed/refractory (R/R) acute lymphocytic leukemia (ALL) [82] (Table 2).

**Table 2:** Clinical trials of CAR-Treg, CAR-γδT, CAR-NKT, CAR-NK, CAR-M and iCARs

	**NCT**	**Cell source**	**Target**	**Cancer**	**Phase**	**Status**	**Location**
**Treg** [3]	NCT05114837	Treg	CD19	ALL	I/II	Not yet recruiting	US
	NCT04817774	Treg	HLA-A2	Renal Transplant Rejection	I/II	Recruiting	Europe
	NCT05234190	Treg	HLA-A2	Liver Transplant Rejection	I/II	Recruiting	UK
**γδT cells** [7]	NCT02656147	γδT	CD19	B-cell Lymphoma, ALL, CLL	I	Unknown	China
	NCT05554939	γδT	CD19	NHL	I/II	Recruiting	China
	NCT04735471	γδT	CD20	B-cell Malignancies	I	Recruiting	US
	NCT04702841	γδT	CD7	T-cell Malignancies	Early I	Recruiting	China
	NCT05388305	γδT	CD123	AML	NA	Recruiting	China
	NCT04796441	γδT	CD123	AML	NA	Recruiting	China
	NCT04107142	γδT	NKG2DL	Solid Tumors	I	Unknown	Malaysia
**NKT cells** [5]	NCT05487651	NKT	CD19	B-cell Malignancies	I	Recruiting	US
	NCT03774654	NKT	CD19	B-cell Malignancies	I	Recruiting	US
	NCT00840853	NKT	CD19	ALL, NHL	I	Active, Not recruiting	US
	NCT04814004	NKT	CD19	B-cell Malignancies	I	Recruiting	China
	NCT03294954	NKT	GD2	Neuroblastoma	I	Active, Not recruiting	US
**NK cells** [48]	NCT00995137	PB NK	CD19	ALL	I	Completed	US
	NCT05020678	PB NK	CD19	B-cell Malignancies	I	Recruiting	US
	NCT03824964	PB NK	CD19/22	B-cell Lymphoma	Early I	Unknown	Unknown
	NCT03692767	PB NK	CD22	B-cell Lymphoma	Early I	Unknown	Unknown
	NCT04623944	PB NK	NKG2DL	AML, MDS	I	Recruiting	US
	NCT03415100	PB NK	NKG2DL	Metastatic Solid Tumors	I	Unknown	China
	NCT03692637	PB NK	Mesothelin	Epithelial Ovarian Cancer	Early I	Unknown	Unknown
	NCT02944162	NK-92	CD33	AML	I/II	Unknown	China
	NCT02742727	NK-92	CD7	Leukemia, Lymphoma	I/II	Unknown	China
	NCT02892695	NK-92	CD19	Leukemia, Lymphoma	I/II	Unknown	China
**NK cells** [48]	NCT03690310	NK-92	CD19	B-cell Lymphoma	Early I	Unknown	Unknown
	NCT03692663	NK-92	PSMA	Prostate Cancer	Early I	Recruiting	China
	NCT03940833	NK-92	BCMA	MM	I/II	Unknown	China
	NCT02839954	NK-92	MUC1	Solid Tumors	I/II	Unknown	China
	NCT03940820	NK-92	ROBO1	Solid Tumors	I/II	Unknown	China
	NCT03931720	NK-92	ROBO1	Malignant Tumor	I/II	Unknown	China
	NCT03941457	NK-92	ROBO1	Pancreatic Cancer	I/II	Unknown	China
	NCT05528341	NK-92	NKG2DL	Solid Tumors	I	Recruiting	China
	NCT03383978	NK-92	HER2	Glioblastoma	I	Recruiting	Germany
	NCT03656705	NK-92	PD-L1	Advanced NSCLC	I	Recruiting	China
	NCT04050709	NK-92	PD-L1	Advanced Solid Cancers	I	Active, Not recruiting	US
	NCT04847466	NK-92	PD-L1	GEJ Cancers, HNSCC	II	Recruiting	US
	NCT04796675	CB NK	CD19	B-Lymphoid Malignancies	I	Recruiting	China
	NCT03056339	CB NK	CD19	B-Lymphoid Malignancies	I/II	Active, Not recruiting	US
	NCT05472558	CB NK	CD19	NHL	I	Recruiting	China
	NCT05667155	CB NK	CD19/CD70	NHL	I	Not yet recruiting	China
	NCT05092451	CB NK	CD70	AML, MDS, B-cell Lymphoma	I/II	Recruiting	US
	NCT05110742	CB NK	CD5	Hematological Malignances	I/II	Not yet recruiting	US
	NCT05008536	CB NK	BCMA	MM	Early I	Recruiting	China
	NCT05673447	Unknown	CD19	DLBCL	Early I	Not yet recruiting	Unknown
	NCT05645601	Unknown	CD19	B-cell Malignancies	I	Recruiting	China
	NCT04639739	Unknown	CD19	NHL	Early I	Not yet recruiting	China
	NCT04887012	Unknown	CD19	NHL	I	Recruiting	China
	NCT05654038	Unknown	CD19	B-cell Malignancies	I/II	Recruiting	China
	NCT05410041	Unknown	CD19	B-cell Malignancies	I	Recruiting	China
**NK cells** [48]	NCT05336409	Unknown	CD19	B-cell Malignancies	I	Recruiting	US
	NCT05563545	Unknown	CD19	ALL	I	Completed	China
	NCT04796688	Unknown	CD19	B-cell Malignancies	I	Recruiting	China
	NCT05652530	Unknown	BCMA	MM	Early I	Recruiting	China
	NCT05008575	Unknown	CD33	AML	I	Recruiting	China
	NCT05215015	Unknown	CD33/CLL1	AML	Early I	Recruiting	China
	NCT05574608	Unknown	CD123	AML	Early I	Recruiting	China
	NCT05247957	Unknown	NKG2DL	AML	NA	Terminated	China
	NCT05213195	Unknown	NKG2DL	Metastatic Colorectal Cancer	I	Recruiting	China
	NCT05507593	Unknown	DLL3	SCLC	I	Recruiting	China
	NCT05410717	Unknown	CLDN6	Advanced Solid Tumors	I/II	Recruiting	China
	NCT05137275	Unknown	5T4	Advanced Solid Cancers	Early I	Recruiting	China
	NCT05194709	Unknown	5T4	Advanced Solid Tumors	Early I	Recruiting	China
**Macrophages** [1]	NCT04660929	Macrophages	HER2	Solid Tumors	I	Recruiting	US
**iPSCS** [5]	NCT04629729	FT819	CD19	B-cell Lymphoma, ALL, CLL	I	Recruiting	US
	NCT05182073	FT576	BCMA	MM	I	Recruiting	US
	NCT04245722	FT596	CD19	B-cell Lymphoma, CLL	I	Recruiting	US
	NCT04555811	FT596	CD19	NHL, DLBCL, B-cell Lymphoma	I	Recruiting	US
	NCT03824951	iPSCs	CD19	B-cell Lymphoma	Early I	Unknown	Unknown

*AML* Acute Myeloid Leukemia, *CLL* Chronic Lymphocytic Leukemia, *NHL* Non-Hodgkin lymphoma, *MM* Multiple Myeloma, *AML* Acute Myeloid Leukemia, *MDS* Myelodysplastic Syndromes, *NSCLC* Non-Small Cell Lung Cancer, *GEJ* Gastric and Gastroesophageal Junction, *HNSCCs* Head and Neck Squamous Cell Carcinomas, *DLBCL* Diffuse large B-cell lymphoma, *SCLC* Small Cell Lung Cancer

#### Limitations

Despite encouraging results, there are disadvantages and limitations associated with CAR-Tregs. Although expression of a CAR gene in Tregs redirects them to the site of inflammation or autoimmune activity, thereby increasing their suppressive efficiency while avoiding systemic immunosuppression, it remains to be determined whether CAR-Tregs would also induce adverse reactions, such as CRS and neuronal cytotoxicity, similar to that of CAR-T cells [47]. Furthermore, exhaustion of CAR-Tregs likely limits their efficacy in suppression. Most CAR-Tregs have used second-generation CARs bearing CD28 costimulatory domain [59]; one should evaluate whether this costimulatory region may render CAR-Treg exhaustion. Finally, like other cell-based products, the production of engineered CAR-Tregs is expensive and requires specialized equipment.

### γδT cells

#### Properties

γδT cells are a subset of innate-like T lymphocytes containing a TCR composed of γ and δ chains [83]. They comprise 0.5–5% of circulating T cells but are the predominant lymphocyte at epithelial surfaces [84]. As their name indicates, γδT cells develop in the thymus, and then some move to the lymph nodes (LNs), while many directly migrate to the periphery or mucosal tissues, where they encounter antigens [85, 86]. Therefore, they show less clonal expansion and TCR diversity than αβT cells, whose clonal expansion and activation occur in the LNs and the T cell zones of the spleen, where they regularly encounter vast numbers of APCs presenting diverse antigens [87]. Human γδT cells are a heterogeneous population that can be divided into a variety of subsets depending on their TCR γ and δ chains. For example, they are classified into four main subpopulations (Vδ1, Vδ2, Vδ3, and Vδ5) based on the type of δ chain they express at their TCRs [83]. Further subsets are formed when these four δ chains are paired with seven different Vγ (Vγ2/3/4/5/8/9/10) chains. Vδ2, which is almost solely paired with Vγ9 to create Vγ9Vδ2, and Vδ1, which is co-expressed with various Vγ, are two main subsets of human γδT cells, each having distinct tissue distribution and antigen-specificities [88]. Vγ9Vδ2T cells are the predominant γδT cell subset in human peripheral blood (50–95%) that recognize microbe-derived and host-derived phosphoantigens (pAgs) presented by butyrophilin 3A1 (BTN3A1) and BTN3A2 [89]. Vδ1 + T cells, on the other hand, reside mainly within epithelial tissues, recognizing different classes of antigens, including lipid-antigens presented by CD1 and stress-inducible MHC class I related-chain antigens (MIC) A/B [90, 91]. At the effector level, Vδ2Vγ9 T cells rapidly produce pro-inflammatory cytokines, including TNF-α and IFN-γ, whereas Vδ1 T cells produce other cytokines, among which insulin-like growth factor-1 (IGF-1), contributing to local wound healing [92, 93]. However, both subsets are endowed with potent anti-tumor cytolytic function, with the latter known as tissue-associated or tumor-infiltrating lymphocytes. The γδT cells serve as a bridge between innate and adaptive immune responses. Their ability to undergo early and rapid expansion in response to infections, inflammation, and tumor, likely due to recognizing families of unprocessed antigens with conserved molecular patterns in an MHC-unrestricted manner, allows γδT cells to function as the first line of defense, together with innate immune cells such as macrophages and neutrophils. However, possessing junctionally diverse TCRs generated by gene rearrangement and their ability to produce a set of cytokines similar to αβT cells, mount cytotoxic responses, and develop memory phenotype upon activation proves that γδT cells are partially adaptive [90, 94]. γδT cells exert their potent anti-tumor activities through different mechanisms and receptors. They secret cytotoxic molecules such as perforin and granzymes or express apoptosis-inducing ligands, such as TNF-related apoptosis-inducing ligand (TRAIL) and Fas ligand (FasL), to directly kill tumor cells [95]. γδT cells also upregulate CD16 (FcγRIIIa) to participate in antibody-dependent cellular cytotoxicity (ADCC) [96]. Furthermore, they express a set of NK receptors (NKRs), including natural-killer group 2 member D (NKG2D), and the natural cytotoxicity-triggering receptors (NCRs), such as NKp30 and NKp44, for tumor recognition and killing [95, 97].

#### Advantages

γδT cells own several favorable characteristics over αβT cells that encourage their clinical applications. First, they can recognize a broad spectrum of antigens shared by a variety of stressed and tumor cells, thus reducing the chances of tumor escape by single antigen loss [98]. Second, γδT cells have potent cytolytic activity against transformed cells. Studies showed that these cells exert strong anti-tumor responses in vitro and in vivo, suggesting natural roles in tumor control and potential for therapeutic exploitation [99]. Third, they can stimulate and regulate the biological functions of other cell types, such as dendritic cells (DCs), CD8 + T cells, and NK cells, thereby enabling the orchestration of a cascade of immune responses against tumors [87]. Forth, γδT cells can recognize their target cells in an MHC-independent manner and do not cause GvHD, thus making them an attractive source for adoptive cell immunotherapy, particularly in allogeneic settings. Fifth, they can be readily expanded to high numbers in vitro and in vivo, especially in the presence of amino bisphosphonates, such as zoledronate (ZOL), the most commonly used agent for activation/expansion of γδT cells in clinical trials [100]. Finally, they, especially the Vδ1 subset, have the natural ability to home in a wide variety of tissues wherein they can rapidly respond to the target antigens and release effector cytokines [87]. A meta-analysis of gene expression data from more than 18,000 cancers revealed that infiltration by γδT cells is the most significant factor associated with favorable prognosis [101]. Moreover, a terminated phase I clinical trial (NCT00562666), in which autologous γδT cells infused in combination with IL-2 into ten patients with metastatic renal cell carcinoma (mRCC), have proved the safety and efficacy of γδT cells in the clinical setting [102]. These unique characteristics have prompted researchers to use γδT cells for CAR generation. However, there are relatively few studies on CAR-γδT cells compared to the substantial body of literature on αβT cells expressing CARs. Arming γδT cells with a CAR may provide a way to use allogeneic (off-the-shelf) CARs safely and to target minor clones with lower antigen density, which may not be eliminated by the CAR-αβT cells [100].

#### Clinical applications

The first study to engineer γδT cells with chimeric receptors was published in 2004 by Rischer et al., demonstrating in-vitro antigen-specific IFN-γ secretion and cytotoxicity against target cells using peripheral blood-derived Vγ9Vδ2 T cells transduced with retroviral vectors encoding either GD2 or CD19-specific chimeric receptors [103]. Later, several studies have examined the anti-tumor efficacy of γδT cells engineered with second-generation scFv-based CARs (mostly 28ζ CARs) directed towards several tumor-specific antigens in the context of both hematological and solid tissue malignancies [83, 100, 104]. Their results revealed that CAR-γδT cells mediate antigen-dependent anti-tumor activity against their respective cancer targets both in vitro and in vivo [83, 100, 104]. An ongoing clinical trial (NCT02656147) using anti-CD19 CAR-γδT cells for hematological malignancies has confirmed the clinical benefit of CAR-engineered γδT cells [100, 105]. In contrast to CD19-specific CAR-αβT cells, CD19-directed CAR-γδT cells have shown reactivity against both CD19-positive and negative tumor cells in vitro and in vivo, an effect that was enhanced by ZOL, suggesting that CD19-directed CAR-γδT cells may target leukemic cells also after antigen loss and retain tumor antigen recognition via their γδTCR [100]. Four more clinical trials are underway to assess the safety and efficacy of CAR-γδT cells targeting other tumor antigens, such as CD20, CD7, and CD123, in hematological malignancies [106, 107] (Table 2). Their natural ability to infiltrate and function in hypoxic environments of tumors makes γδT cell therapy attractive for solid tumors, which have not benefited from conventional CAR-T cells. Accordingly, a clinical trial (NCT04107142) is evaluating the safety and efficacy of NKG2D-based CAR-γδT cells against several relapsed or refractory (r/r) solid tumors, including colorectal cancer (CRC), triple-negative breast cancer (TNBC), sarcoma, nasopharyngeal carcinoma, prostate, and gastric cancers [108]. NKG2D-based CARs use the extracellular domain of the human NKG2D as the antigen recognition domain to target NKG2D ligands (NKG2DL) that are expressed on malignant or stressed cells and typically absent in healthy tissue [109]. In addition to endogenous cytotoxicity against various tumors, Vγ9Vδ2 cells can develop into potent APC (γδT-APC) and acquire phenotypic characteristics of professional APCs, including capacity for cross-presentation of tumor-associated antigens [110, 111]. Capsomidis et al. indicated that γδT cells transduced with second-generation anti-GD2 CARs (GD2-28ζ) retain the ability to cross-present TAAs leading to a clonal expansion of αβT cells [83].

#### Limitations

Despite encouraging results, CAR-γδT cells are associated with drawbacks. Fisher et al. showed that, like αβT cells, γδT cells transduced with GD2-28ζ CAR exhibited an exhausted phenotype after 16 days of culture with IL-2. As tonic signaling was most evident in pathways downstream of CD3ζ, they diminished tonic signaling by modifying γδT cells with chimeric costimulatory receptors (CCRs) lacking CD3ζ but containing DAP10, a costimulatory molecule downstream of NKG2D [112]. However, studies showed that not all populations of γδT cells are prone to CAR-induced exhaustion. Capsomidis et al. revealed that transduction with a tonically signaling second-generation CAR, like GD2-28ζ CAR, led to increased expression of PD-1 and TIM-3 in Vδ2 + , but not Vδ1 + cells, even in the absence of cognate antigen [113].

### Mucosal-associated invariant T cells

#### Properties

MAIT cells are a unique, evolutionarily conserved, innate-like subpopulation of T cells enriched in the liver and mucosal tissues [114, 115]. They are very few at birth and accumulate gradually during infancy, taking at least six years to reach adult levels [116]. MAIT cells express a semi-invariant αβTCR that recognizes non-peptide antigens presented by the non-polymorphic MHC class I-related molecule MR1 [114, 115]. Non-peptide antigens presented by MR1 are 5-(2-oxoethylideneamino)-6-D-ribitylaminouracil (5-OERU) and 5-(2-oxopropylideneamino)-6-D-ribitylaminouracil (5-OP-RU) produced by a wide variety of bacteria, mycobacteria, and yeasts during riboflavin (vitamin B2) synthesis [117, 118]. CD161, a C-type lectin-like receptor or IL-18Rα, plus the Vα7.2 segment, can be used for MAIT cell identification in peripheral blood and tissues [119]. MAIT cells are divided into different subsets based on the expression of the CD4 and CD8 co-receptors. In human peripheral blood, MAITs cells are predominantly CD8 + or double negative (CD4-CD8-). The CD4 + and double positive (CD4 + CD8 +) subsets constitute a small portion of total circulating MAIT cells [120]. MAIT cells can be activated in TCR-dependent and independent manner. As a nonclassical cytotoxic T cell subset, MAIT cells show potent cytotoxic activity by secreting perforin, granzyme B, expressing TRAIL (TNFSF10), and FasL [121] or producing pro-inflammatory cytokines such as IFN-γ, TNF, GM-CSF, and IL-17 to crosstalk with neutrophils, macrophages, and other effector T cells [122]. TCR-dependent activation is also involved in the tissue-repair program. MAIT cells are stimulated by the cytokines IL-7/-12/-15/-18 and type-I IFNs in a TCR-independent manner, allowing them to contribute to antiviral host defense. TCR-dependent activation generally results in a more rapid immune response and production of inflammatory cytokines than cytokine-mediated activation. Although these two responses are different, they work synergistically to provide optimal MAIT cell activation [121]. MAIT cells also express NK activating receptors, including NKG2D, NKp33, and NKp40, to support their cytotoxic capacity [123].

#### Advantages

MAIT cells possess several favorable properties that make them excellent candidates for CAR technology. First, they are abundant in human tissues, representing up to 45% of liver lymphocytes [119]. They are also numerous in human adult blood, constituting up to 10% of circulating T cells, but very few in cord blood [116, 124]. Second, MAIT cells have an intrinsic effector memory phenotype (CD45RA-CD45RO + CD62LlowCD161 +), with the ability to rapidly mount an immune response upon activation [119, 125]. Third, they possess an intrinsic migratory capacity to peripheral tissues attributed to high expression of tissue homing markers (CCR5, CCR6, CCR9, and CXCR6) [119, 122, 126]. Given that MAIT cells predominantly reside in liver and mucosal-associated peripheral tissues such as the lung, gastrointestinal tract, colon, and cervix, cancers that arose in these tissues may likely be more amenable to MAIT cell-based therapy [122]. Finally, MAIT cells show potent cytotoxic activity upon stimulation. Preclinical studies have shown that MAIT cells may have pro-tumor effects, initiating and accelerating tumor growth by suppressing T/NK cell effector functions; however, activation of MAIT cells by riboflavin intermediates converts them from pro-tumor to anti-tumor effectors [127]. Moreover, as MAIT cells are MHC-unrestricted, they are unlikely to induce GvHD, thus holding great potential as a platform for allogeneic immunotherapy development.

#### Clinical applications

Studies showed that CAR-MAITs specific to CD19 and HER2 displayed similar or, in some cases, significantly higher cytotoxicity than their CAR-T counterparts while offering better safety profiles [124]. Another study showed that in a 3D tumor/tumor-associated macrophage (TAM)/T-cell organoid culture used to mimic TME and the immunosuppressive function of TAMs, anti-mesothelin CAR-MAIT cells could successfully retain their anti-tumor potency, probably by direct targeting TAMs via their NK-activating receptors and TCRs [123]. Bohineust et al. demonstrated that anti-CD19 CAR-MAIT cells could engraft without mediating GvHD in xenograft models, unlike their anti-CD19 CAR-T counterparts [128]. Nevertheless, there have been no registered clinical trials testing CAR-MAITs yet.

#### Limitations

MAIT may also be associated with some drawbacks [129]. As the frequency of MAIT cells varies in the peripheral blood of individuals, the CAR-MAIT numbers obtained after ex vivo expansion may be limited. Also, CAR-MAIT may display exhausted phenotypes, as MAIT cells with exhaustion phenotypes have been detected in chronic viral hepatitis [129, 130].

### Natural killer T cells

#### Properties

Natural killer T (NKT) cells, characterized as CD3 + CD56 + , are an innate-like subset of αβT cells that share morphological and functional characteristics with T and NK cells [131]. They develop in the thymus, diverging from conventional T-cell development at the CD4 + CD8 + double-positive (DP) thymocyte stage in the thymic cortex [132]. In contrast to conventional T cells, TCRs expressed on the surface of NKT cells recognize lipid and glycolipid antigens presented by the monomorphic MHC-class I-like molecule, CD1d [133]. NKT cells can be divided into two major subtypes based on their TCR diversity and antigen specificities. Type I NKT cells, also known as invariant NKT (iNKT) cells, employ an invariant TCRα chain (Vα24-Jα18) combined with a limited repertoire of TCRβ chains to recognize the antigen α-galactosylceramide (α-GalCer) bound within the CD1d. Type II NKT cells, on the other hand, expressing more diverse TCRs recognize a wide range of ligands [134]. The iNKT cells, albeit rare, are evolutionarily conserved and the main subset in tumor immunosurveillance [134]. Like γδT cells, NKT cells seem to be in a preactivated state, supplying timely and effective defense several days before the conventional αβT cells proliferate and differentiate into the effectors. Their rapid response puts NKT cells in the very first lines of innate defense against some types of bacterial and viral infections. Besides, many cytokines secreted by NKT cells can promote differentiation and functions of αβT cells, linking NKT cells to adaptive defense [84]. Activated NKT cells can kill target cells (infected and tumor cells) via perforin/granzyme-mediated cytotoxicity and apoptosis-inducing ligands. However, the most important in vivo effector function of activated NKT cells is the secretion of a broad array of cytokine and chemokine, by which they can promote or suppress immune responses. They also express NK receptors, including NKG2D, NKp33, and NKp40 [84]. A part of the NKT cell population also expresses CD16 (FcγIIIa); however, they are not directly involved in ADCC [135, 136].

#### Advantages

iNKT cells own several favorable characteristics that encourage their clinical applications, including [1] potent anti-tumor function through direct cytotoxicity or αβT cell cross-priming [137], [2] natural ability to effectively traffic to the tumor site [138], [3] disrupting the suppressive activity of TAMs and myeloid-derived suppressor cells (MDSCs) in CD1d-dependant manner, [4] recognition and cytotoxic killing of TAMs independently of CD1d via NKRs [106], [5] and no risk of GvHD due to lack of MHC engagement [139, 140]. Although the frequency of iNKT cells is low in the human periphery, the discovery that they can be exponentially expanded ex vivo or in vivo through stimulation with α-GalCer-loaded DCs prompted their application in cancer treatment [141]. Arming iNKT cells with CAR may provide a potent anti-tumor therapy that can remediate the tumor microenvironment (TME) through simultaneous depletion of TAMs and tumor cells using TCR/CD1d and CAR recognition, respectively, as well as generalized elimination of both via NK receptors.

#### Clinical applications

In 2014 Heczey et al. genetically manipulated primary human iNKT cells to express different CAR constructs (without costimulation or with the CD28 and 4-1BB costimulatory domains either alone or in combination) targeting the GD2 ganglioside, which is highly expressed on neuroblastoma cells. They showed that engineered iNKT cells, irrespective of the CAR design, could kill tumor cells through GD2-directed CAR and CD1d-dependent TCRs [142]. Also, CAR constructs containing 4-1BB either alone or combined with CD28 skewed the iNKT-cell cytokine profile toward Th1, as evidenced by increased production of IFN-γ and GM-CSF. In addition, anti-GD2 CAR-NKT cells, irrespective of the CAR design, demonstrated increased tumor homing and killing capacities and, in contrast to anti-GD2 CAR-T cells, did not induce GvHD [142]. However, anti-GD2 CAR-NKT cells suffered from low in vivo persistence. To overcome this limitation, Xu et al. incorporated the gene encoding IL-15, which had previously been shown to protect NKTs from a highly hypoxic TME and reverse tumor immune suppression, into the anti-GD2 CAR expression cassette [143, 144]. In xenografts models of NB, NKT cells co-expressing anti-GD2 CAR and IL15 exhibited remarkable persistence, improved tumor infiltration, and enhanced anti-tumor activity [144]. As NKT cells are a small proportion of T cells in the blood, making up less than 0.1% of T cell populations, their production on a large scale with high purity for clinical use was a critical challenge, which overcame by Heczey and colleagues. They developed a current Good Manufacturing Practice (cGMP) protocol for NKT cell isolation and expansion, by which they achieved a mean NKT purity and CAR expression of 96% and 54%, respectively [145]. These studies paved the way for launching the first-in-human CAR-NKT clinical trial evaluating the safety of anti-GD2 CAR and IL-15 expressing autologous NKT cells (NCT03294954) in patients with relapsed/ resistant neuroblastoma [145]. Initial results of this clinical trial show that treatment is safe in the ten patients enrolled, with one complete response, one partial response, and three patients with stable disease [145]. In another study, Tian et al. genetically engineered primary human iNKT cells with anti-CD19 CAR to use in B-cell malignancies. They evaluated the capacity of NKT cells with a memory phenotype (CD62L +) to induce prolonged in vivo persistence [146]. They found that compared to CD62L-iNKT cells, CD62L + iNKT cells exhibited 8-fold greater in vitro expansion in response to antigenic stimulation and significantly longer in vivo persistence without exhaustion and cell death.

Furthermore, in xenograft models, only CD62L + iNKT cells expressing anti-CD19 CAR induced sustained tumor regression and enhanced survival [146]. Further studies revealed that anti-CD19 CAR-NKT cells are more effective than anti-CD19 CAR-T cells against CD1d-expressing lymphomas in vitro and in vivo, as they can eliminate CD1d + tumor cells by dual targeting of CD19 and CD1d on target cells, suggesting that anti-CD19 CAR-NKT cells may provide better therapeutic outcomes than traditional CAR-T cells in these tumors types [147]. Four clinical trials are currently underway, examining the safety and efficacy of CD19-specific CAR-iNKT cells modified to secrete IL-15 in relapsed and refractory B-cell malignancies [107] (Table 2). CAR-NKT cells targeting chondroitin sulfate proteoglycan-4 (CSPG4), CD38, and the plasma cell-specific B cell maturation antigen (BCMA) have also been developed and showed CAR-specific as well as TCR-dependent cytotoxicity against melanoma cells [141, 148].

#### Limitations

Despite promising results of iNKT cells in preclinical and clinical studies, several associated limitations hinder the application of these cells. For example, their low frequency in human peripheral blood makes it difficult to grow large numbers of iNKT cells for CAR engineering. Therefore, the initial cell materials require optimized expansion protocols, usually involving agonists, such as α-GalCer-loaded feeder cells and cytokines, followed by enrichment, purification, and subsequent cell engineering [149, 150].

### Natural killer cells

#### Properties

Natural killer (NK) cells are large granular lymphocytes of innate immunity that predominantly circulate in the blood, making up 5–10% of human peripheral blood lymphocytes [151]. They can be distinguished from T and iNKT cells by their lack of CD3 and TCRs [152]. NK cells often show surface expression of CD56 and CD16 (FcγIIIa) that can be used for their identifications in the periphery [84]. NK cells were initially defined in the 1970s by Kiessling and Herberman, who concurrently described them as a distinct subpopulation of lymphocytes able to recognize and eliminate tumor cells without prior encounter [153, 154]. They are originated from common lymphoid progenitor (CLP) cells in the bone marrow (BM) and undergo complete maturation in secondary lymphoid tissues [155]. During development, they are functionally tuned to self-MHC class I under an adaptive process termed education. NK cells can be divided into two main subgroups, including CD56^dim^ and CD56^bright^ cells, based on the CD56 expression level on their surface [156]. CD56^dim^ NK cells, which account for 90% of circulating NK cells, are developmentally more mature with higher cytotoxic activity against tumor cells than the CD56^bright^ subset. The CD56^bright^ NK cells, on the other hand, reside predominantly in the lymphoid organs and possess a greater capacity to produce cytokines and chemokines [157]. The stimulation and regulation of NK cell function are mediated by an array of activating and inhibitory surface receptors (Table 3). The interaction of activating receptors with stress- or virus-related ligands on target cells triggers NK cell activation under a mechanism known as induced-self recognition. NK cells also display a potent missing-self response to target cells that downregulate or lose surface expression of MHC class I molecules to evade T cell anti-tumor immunity [158]. In humans, the key NK activating receptors involved in target cell killing are the natural cytotoxicity receptors (NCRs; NKp44, NKp30, NKp46) and NKG2D [159]. In addition to activating receptors, NK cells can exert their anti-tumor activity via several mechanisms, including perforin/granzyme-mediated natural cytotoxicity, FcγIIIa-mediated ADCC, FasL, and TRAIL [160].

**Table 3:** Examples of NK activating and inhibitory receptors

**NK Activating Receptors**	**Receptor Class**
NKp46	NCR
NKp30	NCR
NKp44	NCR
NKG2D	CD94/NKG2
NKG2C	CD94/NKG2
KIR2DS1	KIR
**NK Inhibitory Receptors**	**Receptor Class**
NKG2A	CD94/NKG2
NKG2B	CD94/NKG2
KIR3DL1	KIR
KIR3DL2	KIR
KIR2DL1	KIR
KIR2DL2	KIR
KIR2DL3	KIR
KIR2DL4	KIR

*NCRs* Natural Cytotoxicity-Triggering Receptors, *KIR* Killer Cell Ig-Like Receptors

#### Advantages

As the main effector cell type of innate immunity, NK cells offer several advantages over T cells, making them an attractive candidate for cell-based immunotherapy. First, they are rapid responders able to target tumors without pre-sensitization. Second, as mentioned above, NK cells can eliminate target cells through various mechanisms. They can also secrete multiple cytokines and chemokines to modulate the function of other innate and adaptive immune cells [161]. Third, while tumor escape via decreased MHC-I expression renders CAR-T cells helpless in detecting tumor cells, it can sensitize them to NK cell-mediated lysis via self-missing recognition [162]. Forth, trials using adoptive transfer of allogeneic NK cells demonstrate that administration of these cells is safe and well-tolerated with little evidence of toxicities such as CRS, neurotoxicity, or GvHD [163, 164]. Studies showed that in HLA-incompatible murine models of hematopoietic stem cell transplants (HSCT), donor NK cells not only cause no GvHD, they even exert a protective effect against GvHD by destroying the recipient’s antigen-presenting cells. Accordingly, this effect could promote engraftment and make allogenic NK cells less susceptible to immune rejection [165]. Finally, as no serious adverse effects are observed or expected with CAR-NK cells, infusion of modified NK cells and follow-up of the disease status can be performed in an outpatient environment, significantly reducing the indirect costs associated with CAR-based immunotherapy. Furthermore, the possibility of using off-the-shelf allogenic NK cells would offer a further advantage in terms of time and cost as allogeneic cell therapy products can be manufactured in advance and readily available for multiple patients on demand. The potent anti-tumor activity and the favorable safety profile of NK cells make them an attractive platform for CAR by which their anti-tumor potential is redirected towards specific antigens. In addition to PBMCs, NK cells can be obtained from various sources, such as UCB, BM, NK cell lines, and iPSCs. Human embryonic stem cells (hESCs) and HSPCs also serve as sources of NK cells [151, 166]. As isolation, purification, ex-vivo expansion, and transduction of primary NK cells are challenging, NK-92, an IL-2-dependent human NK cell line, is a common NK source for CAR-NK generation due to its infinite proliferative capacity, high endogenous cytotoxic potential, and phenotypic and functional characteristics analogous to activated NK cells. Moreover, they are easy to transduce and do not carry any contaminating T cells to cause GvHD [167].

#### Clinical applications

There has been an accumulation of preclinical data indicating that CAR-modified NK-92 cells have promise in the treatment of hematological malignancies such as lymphoma (anti-CD19 and anti-CD20 CARs) [168], acute myeloid leukemia (anti-CD33 CAR) [169], multiple myeloma (anti-CS1 CAR) [170], T-cell acute lymphoblastic leukemia (anti-CD7 CAR) [171], peripheral T-cell lymphomas (anti-CD4 CAR) [172], and solid tumors such as breast cancer (anti-HER2 CAR) [173], prostate cancer (anti-PSMA CAR) [174], neuroblastoma (anti-GD2 CAR) [175], glioblastoma (anti-ErbB2 CAR) [176], and gastric cancer (anti-mesothelin CAR) [177]. Clinical studies have also confirmed the feasibility, safety, and potent anti-tumor activity of CAR-NK-92 cells [107, 178, 179] (Table 2). However, there are also several disadvantages associated with NK-92 cells. First, given their malignant nature, CAR-NK cells derived from the NK-92 cell line must be subjected to lethal radiation before the infusion to avoid the risk of secondary tumorigenicity [180, 181]. While having no impact on their cytotoxic potential, phenotype, and functionality, irradiation diminishes in vivo expansion potential of engineered NK-92 cells, thus necessitating multiple injections [174]. Second, the NK-92 cell line carries Epstein–Barr virus and has an abnormal genome [182]. Third, as NK-92 cells lack expression of FcγRIIIa (CD16), they cannot mediate ADCC. These drawbacks prevent NK-92 cells from being an ideal cell source for most CAR-NK therapy approaches [183]. Another NK cell source could be UCB, as NK cells make up 30% of their lymphocytes. UCB NK cells can be readily collected and cryopreserved, thus having the potential for use as “off-the-shelf” therapeutic products. UCB NK cells are contaminated with a few T cells, which are immature and unlikely to induce GvHD. Compared to PB NK cells, UCB NK cells have a better proliferation capacity; however, they are naive in phenotype and function with low cytolytic activity and limited ADCC. Nonetheless, there are several ongoing clinical trials with UCB-derived CAR-NK cells in hematological malignancies [184, 185] (Table 2). Overall, the number of CAR-NK clinical trials has rapidly increased in recent years, with multiple targets being explored in hematological (CD5, CD7, CD19, CD22, CD33, CLL1, BCMA, CD70, CD123) and solid (NKG2DL, HER2, MUC1, PSMA, Mesothelin, ROBO1, DLL3, CLDN6, 5T4, PD-L1) malignancies (Table 2).

### Macrophages

#### Properties

Macrophages are long-lived phagocytic cells of innate immunity that populate all normal tissues, usually in sites where they are most likely to encounter foreign entities [186]. They predominantly arise in the bone marrow from common myeloid progenitor (CMP) that develop into monocytes, which then move into the bloodstream and circulate throughout the body. Monocytes constitute 1–6% of total leukocytes in healthy peripheral blood. After crossing the walls of capillaries into connective tissue, monocytes turn into macrophages [187]. Although macrophage phenotype is plastic and can change in response to cytokines, pathogen-associated molecular patterns, metabolic cues, cell–cell interactions, and tissue-specific signals [188], they are traditionally divided into two subsets: classically activated (M1) and alternatively activated (M2) macrophage. M1 macrophages, typically induced by Th1 cytokines, secrete pro-inflammatory cytokines such as TNF, IL-1β, IL-6, IL-12, and IL-23. They also produce high levels of reactive oxygen species (ROS), reactive nitrogen species (RNS), and the inducible nitric oxide synthase (iNOS) enzyme, which produces the powerful antimicrobial agent nitric oxide (NO). As a result, M1 macrophages promote a highly microbicidal environment and mediate the destruction of pathogens and tumor cells [187, 189]. In cancer, macrophages often adopt the M2 phenotype, the anti-inflammatory phenotype. M2 macrophages secrete immunoregulatory cytokines such as IL-4, IL-10, IL-13, and TGF-β and are involved in tissue repair and remodeling that can collectively support tumor growth, angiogenesis, and invasion [190]. Tumor-associated macrophages (TAMs) are a distinct subpopulation of macrophages residing in the TME, where they promote invasion and angiogenesis, facilitate metastasis, and increase immunosuppression [191]. TAMs are differentiated from bone marrow-derived monocytes actively recruited to the TME via chemoattractants such as CCL2 [192, 193]. They constitute up to 50% of the cell mass within the TME of most solid tumors. Within the TME, hypoxia and elevated Th2 cytokine levels polarize TAM into M2 phenotypes to support tumor progression and metastasis [194]. In addition to potent phagocytic and cytotoxic capabilities, macrophages can initiate and potentiate an adaptive immune response via T-cell recruitment, antigen presentation, co-stimulation, and cytokine secretion [195]. They can also influence surrounding immune cells in both pro- and anti-inflammatory manners and are adept at remodeling the extracellular matrix (ECM) [196]. In addition to FcRs by which macrophages participate in ADCC, they highly express Toll-like receptors (TLRs), the key pattern recognition receptors (PRRs) in mammals. Interaction TLRs with corresponding ligands can stimulate phagocytosis, cellular activation, and the production of pro-inflammatory cytokines in macrophages. TLR engagement on macrophages also triggers the synthesis of iNOS [84].

#### Advantages

The ability to penetrate the TME, perform phagocytosis, present antigens, release pro-inflammatory cytokines, and interact with other immune cells in the TME, make macrophages attractive platforms for CARs [197].

#### Clinical applications

Endowing macrophages with tumor antigen-specific CARs redirects their phagocytic function and results in a targeted anti-tumor therapeutic effect with the potential to stimulate an adaptive immune response [198]. The core components of CAR-M are similar to that of CAR-T; however, the choice of signaling domain is of particular interest when designing CAR-M. Morrissey et al. screened a library of known murine phagocytic receptors to identify an appropriate intracellular signaling domain capable of inducing phagocytosis. They found that CAR constructs containing the multiple EGF-like-domains 10 (Megf10) or FcRγ could effectively trigger phagocytosis [199]. They observed that although most anti-CD19 CAR^Megf10^ (78%) and CAR^FcRγ^ (85%) macrophages could ingest bites of Raji cells within 90 min, whole-cell phagocytosis by CAR-Ms was rare. To enhance the frequency of whole-cell engulfment, they created a ‘tandem’ CAR by fusing a portion of the CD19 cytoplasmic domain responsible for recruiting the p85 subunit of PI3K to CAR^FcRɣ^. They realized that although expression of ‘tandem’ CAR resulted in a three-fold increase in whole-cell eating, other CAR-Ms could efficiently perform whole-cell phagocytosis when co-cultured with CD19 + target cells for two days [199]. As immunosuppressive cells within tumor tissues use CCR7 to migrate to distal immune organs and promote tumor progression, Niu et al. engineered a family of CAR-Ms targeting CCR7 to disrupt this signal transmission. Accordingly, the natural ligand of CCR7, CCL19, was utilized as the extracellular recognition domain. Anti-CCR7 CAR-Ms differed in the intracellular signaling domain, containing activation domains from MerTK, TLR2, TLR4, TLR6, and the 4-1BB-CD3ζ. Anti-CCR7 CAR-Ms bearing the MerTK activation domain exhibited the highest tumor cell toxicity [200]. However, Morrissey et al. observed that anti-CD19 CAR-M bearing the same cytosolic domain (anti-CD19 CAR-M^MerTK^) did not bind antigen-functionalized beads [199]. Such discrepancies warn that optimization and careful functional evaluation is required when developing new CAR-M architectures. Zhang et al. used CD147 as the intracellular signaling domain of an anti-HER2 CAR-M to degrade the tumor extracellular matrix to overcome physical barriers. They observed that CAR-147 triggers the secretion of matrix metalloproteinase (MMPs) within the tumor upon antigen binding without affecting other functions of macrophages [201]. Liu et al. compared three common engulfment receptor intracellular domains (FcRγ, Megf10, and PI3K recruitment domain of CD19) in CAR-M for their phagocytosis and killing ability. CAR-M^FcRγ^ showed the most potent phagocytic and killing capacity among the three CAR-Ms [202]. They also found that CAR-M and CAR-T have a synergistic effect against tumors. The synergistic effect of CAR-M and CAR-T against tumor cells probably depends on a feedback loop triggered by the activation of CAR-T. The inflammatory factors secreted by CAR-T augment the cytotoxicity of CAR-M by inducing macrophage M1 polarization and increasing the expression of costimulatory ligands on CAR-M, which may promote the fitness and activation of CAR-T cells in turn [202]. Klichinsky et al. also demonstrated that CAR-Ms constructed with either the CD3ζ or FcRγ activating domain are functionally similar in phagocytosis assays [198].They generated CAR macrophages (CAR-Ms) by transducing the monocyte-derived pro-inflammatory macrophages with adenoviral vectors encoding anti-HER2 CAR. Their anti-HER2 CAR-Ms demonstrated antigen-specific phagocytosis and tumor clearance in vitro. Also, their CAR-Ms rendered targeted cancer cell phagocytosis while sparing normal cells, decreased tumor burden, and prolonged survival in xenograft models [198]. Characterization of CAR-M activity revealed that CAR-Ms repolarized TAMs toward a pro-inflammatory M1 phenotype, sustained the M1 phenotype, upregulated antigen presentation machinery, recruited and stimulated T and NK cells, and countered the immunosuppressive cytokines [198]. These results led to the first-in-human phase I clinical trial (NCT04660929) to assess the safety, efficacy, tolerability, cell manufacturing feasibility, and trafficking of this CAR-M in subjects with locally advanced or metastatic solid tumors overexpressing HER2 who have failed available therapies [203].

#### Limitations

Although CAR-M has a high potential to become a potent cancer immunotherapy, it is still at its nascent stage, with many problems that need to be overcome to achieve the desired results. First, all previous studies have reported the successful use of first-generation scFv-based CARs to efficiently redirect macrophages, guiding antigen-dependent phagocytosis, cytokine release, and anti-tumor activity; however, CAR macrophage structure needs to be optimized to improve their phagocytosis capacity and crosstalk with T cells. The tandem fusion of the PI3K recruitment domain of CD19 to CAR^FcRγ^ created by et al. Morrissey that tripled the phagocytosis of whole cells can prove the benefit of this structural optimization [199]. Second, gene transfer into primary human macrophages has been a longstanding challenge, as macrophages and monocytes have intrinsic resistance to genetic manipulation [198]. Besides, macrophages do not expand either in vitro or after injection in vivo. Repeated infusions may be needed to maintain sufficient CAR-M levels for active cancer surveillance; however, to solve the two problems simultaneously, cell sources, such as iPSCs or primary human hematopoietic stem/progenitor cells (HSPCs) derived from cord blood, can be used to generate functional CAR-M in sufficient quantities [204, 205]. Third, although an advantage of macrophages over T cells is their homing and infiltrating capacity into tumors, cytokines within TME may repolarize the pro-inflammatory M1 to anti-immune M2 phenotypes, promoting tumor growth and metastasis [206]. Klichinsky et al. showed that the adenoviral vector (Ad5f35) overcame the challenges of genetic manipulation of macrophages and imparted a sustained M1 phenotype [198]; however, more studies need to explore differentiation and retention of the M1 phenotype.

### Neutrophils

#### Properties

Neutrophils, comprising 50–70% of circulating leukocytes and characterized by CD11b + CD16 + CD66b + [207], play a fundamental role in the innate immune response, acting as the first line of defense. Neutrophils are also accumulated in many types of tumors, constituting a significant portion of tumor-infiltrating cells [208]. They originate from myeloid progenitors in the bone marrow, where they become fully differentiated [209]. Produced in the BM at the remarkable rate of 1 × 10^11^ per day, neutrophils constantly patrol the organism for signs of infections and finally die within one or two days [210]. Mature neutrophils are usually retained in the BM via the CXCR4/CXCL12 interaction. Their release into the bloodstream and homing into inflammation sites occur in a chemokine-dependent manner. They destroy pathogens via different mechanisms, including phagocytosis, degranulation, production of ROS, and extracellular traps (NETs), commonly known as NETosis [211, 212]. Neutrophils are also capable of eliminating antibody-coated tumor cells via trogoptosis. Trogoptosis is neutrophil-mediated ADCC, in which neutrophils directly and actively tear off the target cell plasma membrane upon synapse formation, leading to necrotic cell death. Trogoptosis can be further promoted by targeting CD47-SIRPa interactions, which inhibit neutrophil ADCC towards cancer cells [213]. They also express TRAIL, FasL, and pro-inflammatory cytokines such as IFN-γ, TNF, GM-CSF, and so forth [214].

#### Advantages

Given their similarity to macrophages and shared innate anti-tumor response, neutrophils may also present enhanced tumoricidal activities after CAR engineering. Their ability to kill through alternative mechanisms, such as NETosis, is conceptually appealing.

#### Clinical applications

In 1998, before CAR-T cells had gained significant momentum, Roberts and colleagues reported that neutrophils engineered to express an HIV-specific chimeric immune receptor (CIR) containing a CD3ζ intracellular domain showed improved cytotoxicity against tumor cells transfected with the HIV envelope [210]. However, there have been no registered clinical trials testing CAR-specific neutrophils yet.

#### Limitations

As neutrophils are resistant to genetic modification and have a short lifespan with a circulating half-life of 6–8 h [215], other cell sources such as iPSCs and HSPCs need to be engineered with CAR constructs and then differentiated into neutrophils [216, 217].

### Hematopoietic stem/progenitor cells

#### Properties

Hematopoietic stem/progenitor cells (HSPCs), defined as CD34 + cells, are critical for the lifelong maintenance of hematopoiesis through self-renewal and differentiation into mature blood cell lineages [218]. They constantly egress out of the bone marrow (BM) into the blood under homeostatic conditions. They monitor peripheral organs and can foster the local production of tissue-resident innate immune cells under both steady-state conditions and in response to inflammatory signals [219]. Several sources of HSPCs are available for isolation, ex vivo manipulation, and potential re-transplantation, that include BM, peripheral blood following G-CSF stimulation, or cord blood (CB) [204, 220].

#### Advantages

The Modification of human HSPC with CAR provides long-term maintenance of antigen-specific cells of multiple hematopoietic lineages [221]. Transduced HSPCs will produce granulocytes and monocytes within 1–2 weeks, followed by the production of NK cells in a few months and T-lymphocytes potentially for a longer time [222, 223]. CAR expression by these cells may grant persistent anti-tumor immunity with a constantly generated mix of effector cell types, contrary to the current cancer immunotherapy approach using modified mature T-lymphocytes. The production of CAR-modified myeloid and NK cells is attractive as these are the first cells to be produced after HSPC transplantation, becoming the initial effectors until CAR-modified T-cells arise from the thymus, augmenting the graft-versus-tumor (GVT) activity [223]. The introduction of CAR-modified HSPCs in the hematopoietic stem cell transplantation (HSCT) context, which is the standard of care for high-risk patients with CD19-positive hematological malignancies, would favor effective immune response against minimal residual malignancy, support the engraftment of modified cells, and reduce the possibility of immunogenicity of the CAR constructs on the surface of effector cells [223].

#### Clinical applications

De Oliveira et al. transduced CB-derived HSPCs with anti-CD19 CAR and then differentiated them into myeloid or NK cells. They observed that myeloid cells produced from anti-CD19 CAR-modified HSPC showed specific cytotoxicity against CD19-positive tumor cells in vitro [223]. In vivo experiments also demonstrated that CAR-HSPCs could produce multilineage CAR-modified cells that could be detected in bone marrows, spleens, and peripheral blood of xenograft models. In another study, Zhen et al. modified HSPC with HIV-specific CAR to provide long-lived and renewable immunity capable of continuously generating anti-HIV cells. They found that engineering human HSPCs with an HIV-specific CAR can allow the differentiation of HIV-specific T cells and cells of other lineages capable of lowering viral loads in vivo [221]. As the amount and functionality of circulating monocytes of cancer patients may be impacted by previous therapy, thus preventing adequate monocyte-apheresis needed for CAR-M generation, Paasch et al. used CB-derived HSPCs as an alternative cell source to generate functional CAR-M ex vivo [204]. Their results showed that HSPC-derived Ms have typical macrophage morphology, phenotype, and basic anti-bacterial functionality. The generated CAR-Ms targeting the carcinoembryonic antigen (CEA), overexpressed in various solid tumors, showed potent phagocytic activity and cytokine secretion upon antigen recognition [204]. However, there have been no registered clinical trials testing CAR-specific HSPCs yet.

#### Limitations

One concern regarding CAR-modified HSPCs is that the presence of CAR in HSPCs and their progeny cells may activate effector cells in an antigen-independent manner, creating a nonspecific and potentially detrimental pathway for cellular damage [224].

### Induced Pluripotent Stem cells (iPSCs)

#### Properties

iPSCs, in contrast to embryonic stem cells (ESCs), which are derived from the inner cell mass of the blastocyst, can be generated from various mature somatic cells such as skin, fibroblasts, and PBMCs by introducing reprogramming factors (Oct4, Sox2, Klf4, and c-Myc) [225–227].

#### Advantages

**Fig. 1:**
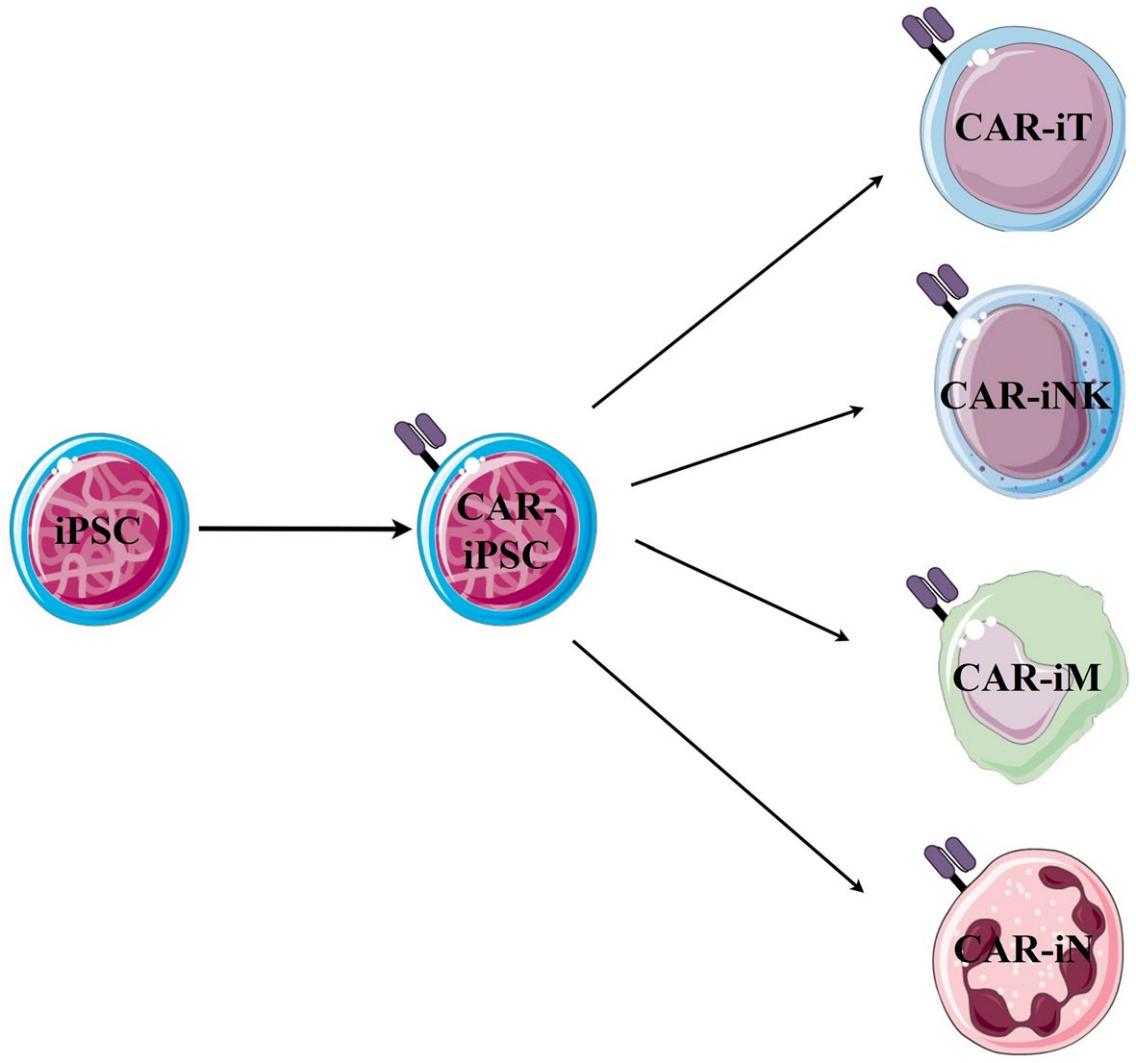
Schematic representations of iPSCs as initial cell source for CAR-cell products generation. M: macrophage, N: neutrophil. The figure was partly generated using Servier Medical Art, provided by Servier, licensed under a Creative Commons Attribution 3.0 unported license

As iPSCs can be propagated limitlessly and differentiated into nearly any specialized cell type [228], it can be an ideal alternative starting material, particularly for those suffering from limited ex-vivo expansion or intrinsic resistance to genetic manipulation or transduction (Fig. 1).

#### Clinical applications

One way to generate an unlimited supply of T cells for CAR technology that establishes long-term immunological memory, and to avoid exhaustion and differentiation-associated senescence, which inevitably arise during in vitro expansion of primary T cells, is to use human induced pluripotent stem cells (iPSCs) as a starting material [229]. The iPSCs typically bear germ-line αβTCR loci that undergo random rearrangements during lymphoid differentiation, thus generating polyclonal T cells of undetermined specificity and HLA restriction [23]. This broad repertoire of unpredictable and antigen-unexperienced TCR severely restricts the effectiveness and potential for expansion and functional characterization of T cells derived from iPSCs. Studies demonstrated that iPSCs developed from T cells with known antigen specificities preserve rearranged TCR genes. Following re-differentiation to T cell lineage, the iPSC-derived T cells (iT) re-expressed the same TCR as the parental T cell. However, with the CAR technology, the antigen specificity of iPSC-derived T cells has been granted [230].

**Fig. 2:**
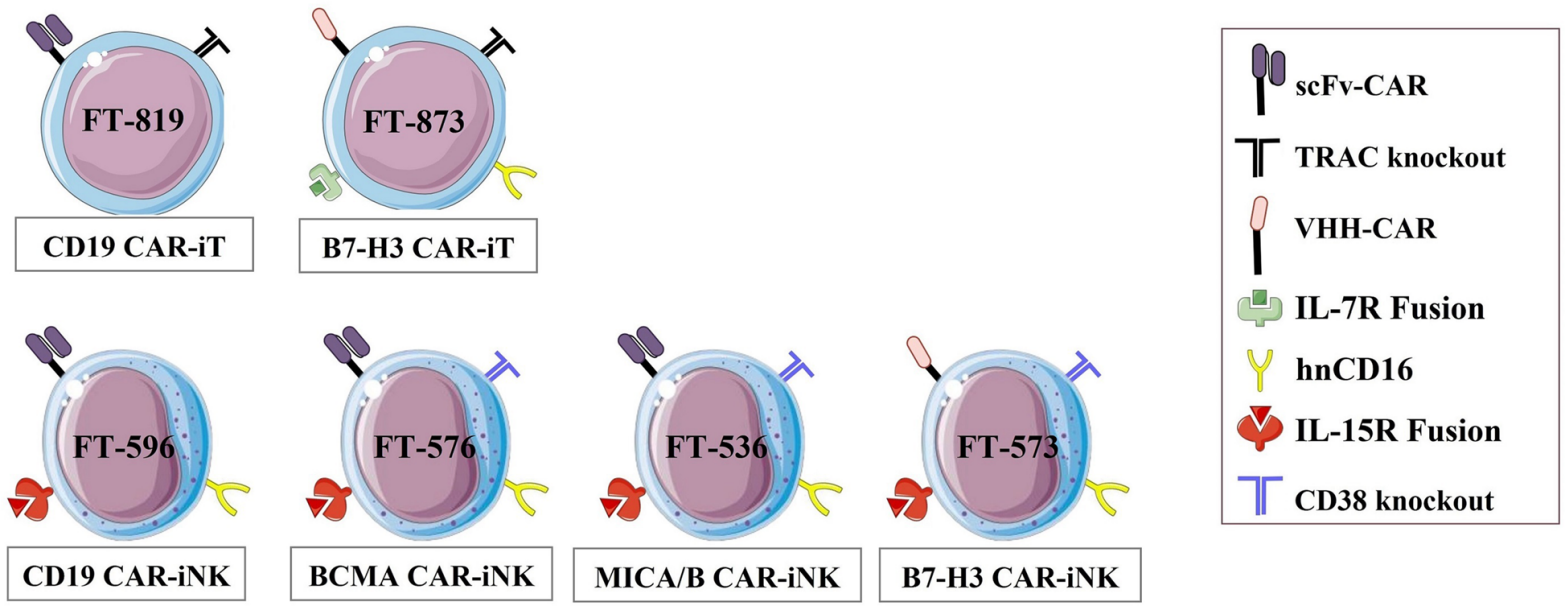
Schematic representations of CAR-cell products of Fate Therapeutics. The figure was partly generated using Servier Medical Art, provided by Servier, licensed under a Creative Commons Attribution 3.0 unported license

Initial efforts to generate antigen-specific CTLs from iPSCs have failed since the reprogrammed CTL are mainly of the CD8αα + homodimers, not CD8αβ heterodimers, and have less antigen-specific cytotoxic activity than primary CTL [230, 231]. In 2013, Themeli et al. generated CD19-specific CAR-T cells derived from iPSCs, where the regenerated T cells were of the CD8αα type (TCRαβ + TCRγδ-CD8α + CD8β-/lowIL2RB-CCR7-CD62L-), being considered to be similar in function to γδT cells [230, 232]. In 2016, Maeda et al. proposed that the failure of CD8αβ T-cell production using the previous methods might be due to the killing of double-positive cells by the double-negative cells in the mixed cultures. Thus, by stimulating purified iPSC-derived CD4/CD8 double-positive cells with anti-CD3 antibody, they generated CD8αβT cells with improved antigen-specific cytotoxicity compared with CD8αα + CTL [233]. Recently Jing et al. demonstrated that knockdown of the histone methyltransferase EZH1, a negative regulator of lymphoid potential during embryonic hematopoiesis, facilitates in vitro differentiation of iPSCs into canonical αβT cell lineages. They observed that when genetically modified with CARs, EZH1-deficient T cells exhibit potent anti-tumor activities in vitro and in vivo [234]. In another study, Wang et al. used artificial thymic organoids (ATOs) to efficiently confer differentiation of iPSCs reprogrammed from CD62L + naive and memory T cells to conventional αβT cell lineages while maintaining CAR expression and functionality. They observed that anti-CD19 CAR-iT cells generated with this method mimic conventional CAR-αβT cells in antigen-specific activation, degranulation, cytotoxicity, and cytokine secretion and effectively control the progression of human CD19 + leukemia in an animal model [235, 236]. In Fate Therapeutics, researchers generate clonal master iPSC lines as renewable sources for the mass production of immune effector cells to provide uniformly engineered, homogenous cell therapy product that is available on-demand for broad patient access [237]. Briefly, they first transduce T cell-derived iPSCs with an expression cassette containing a CAR construct. After single-cell sub-cloning, they screen each engineered iPSC clone for multiple critical quality attributes, including pluripotency, identity, genomic stability, cassette integration, on/off-target integration, and so forth, to select an ideal single-cell-derived engineered iPSC clone to use as the clonal master iPSC line and convert it into a master cell bank [237]. Using this technology, the company developed two CAR-iT products, FT819 and FT873 [238]. In FT819, a novel anti-CD19 CAR bearing a CD28 costimulatory domain and a modified CD3ζ signaling domain is inserted into both alleles of the TRAC gene to achieve uniform CAR expression and eliminate the possibility of GvHD by nullifying the TCR. FT819 product exhibited promising results in-vitro and in the xenograft models of B-cell acute lymphoblastic leukemia (B-ALL) and currently is the first-ever iPSC-derived T-cell therapy to undergo clinical investigation (NCT04629729) [238]. FT873 incorporates three genetically encoded functional attributes, including [1] a VHH-based CAR targeting B7 homolog-3 protein (B7-H3), [2] a high-affinity, non-cleavable CD16 (hnCD16) that maximizes the cytotoxicity of CAR-T cells by allowing co-engagement of CAR and ADCC pathways, and [3] an IL-7 receptor fusion (IL-7RF) that promote proliferation, persistence and homing CAR-iT to TME. Like FT819, in FT873, the single tricistronic expression cassette is inserted into TRAC (Fig. 2). FT873 showed superior tumor control and an increased abundance of tumor-infiltrating effector cells compared to control groups [239].

In another study, Ueda et al. also derived and expanded CD8αβ cytotoxic CAR-T cells from a single human iPSC clone bearing a CAR, aiming to develop long-lasting antigen-specific T-cell immunotherapies against solid tumors. They further modified hiPSC-derived CAR-T (CAR-iT) cells with diacylglycerol kinase (DGK) knockout to enhance the CD3ζ-mediated signal pathway and proliferation of CAR-iT cells as well as with genes encoding for membrane-bound IL-15 and its receptor IL-15Rα (IL-15/IL-15Rα) to improve the persistence and anti-tumor effect of CAR-iT cells. They observed that in multiple tumor-bearing animal models, their CAR-iT cells led to therapeutic outcomes similar to those of primary CD8 αβT cells bearing the same CAR [240].

While many protocols used for generating T cells from iPSCs are somewhat deemed inefficient, the production of iPSC-derived NK (iNK) cells is a routine activity. The iNK cells express typical NK cell surface markers, such as CD56, CD16, NKp46, and NKG2D [241, 242], and exhibit cytotoxicity against diverse target cells via secretion of lytic granules containing perforins and granzymes, production of pro-inflammatory cytokines IFN-γ and TNF-α, and direct cell contact-mediated apoptosis through TRAIL and Fas-FasL interaction [243]. In 2018, Li et al. found that the CAR constructs with an optimal design for NK cells, consisting of the NKG2D transmembrane domain, the 2B4 co-stimulatory domain, and the CD3ζ signaling domain, may confer potent anti-tumor activity in NK cells. Accordingly, their mesothelin-directed NKG2D-2B4ζ CAR-iNK demonstrated a typical NK cell phenotype with enhanced in vivo cytotoxicity, improved mouse survival, and fewer adverse events than CAR-T cells in an ovarian cancer xenograft model [244]. In 2020, Ueda et al. transduced NK/innate lymphoid (ILC) cells derived from HLA homozygous iPSC with a third-generation CD28/4-1BB/CD3ζ CAR targeting glypican 3 (GPC3). The CAR-iNK/ILC showed potent anti-tumor activity in a GPC3-expressing ovarian cancer xenograft model with no risk of general or acute toxicity such as GvHD [245]. In another study, iNK cells engineered to express an epidermal growth factor receptor (EGFR)-CAR demonstrated anti-tumor activity in models of glioblastoma multiforme (GBM) [241]. Besides CAR-iTs, Fate Therapeutics is also developing a set of off-the-shelf CAR-iNK products, including FT596, FT576, FT536, and FT573. FT596 incorporates an NKG2D-2B4ζ CAR targeting CD19 that enhances anti-tumor activity against CD19-expressing B cells, an hnCD16, and a recombinant fusion of IL-15 and IL-15 receptor alpha (IL-15RF) for cytokine-autonomous persistence. Two registered phase I clinical trials are currently evaluating FT596 in B-cell malignancies (Table 2). The FT596 exerts a deep and durable response due to greater degranulation and cytokine release [246]. Like FT596, FT576 contains hnCD16 and IL-15RF but consists of BCMA-specific CAR targeted to the CD38 locus to achieve bi-allelic CD38 knockout to promote persistence under oxidative stress and avoid NK cell fratricide. FT576 demonstrated enhanced efficacy and persistence in-vitro and in a xenograft model of myeloma [247]. Currently, a phase I clinical trial is testing the safety and efficacy of FT576 in patients with multiple myeloma (NCT05182073). Like FT576, the FT536 and FT573 products incorporate four functional modifications: hnCD16, IL-15RF, biallelic CD38 knockout, and a CAR targeting different tumor antigens. FT536 contains a novel CAR that ubiquitously targets the conserved α3 domain of MICA and MICB [248], while FT573 uses the VHH-based CAR similar to that of FT873 [248] (Fig. 2).

Despite promising results achieved by CAR-macrophages (reviewed in the corresponding section), the generation of engineered macrophages remains challenging, mainly due to their limited expansion capacity and general resistance to genetic modifications [249, 250]. Thus differentiation of genetically modified iPSCs to macrophages may offer a more feasible way to develop macrophages with anti-tumor capacity [251]. First iPSCs-derived macrophages (iMs) genetically modified with CAR were generated in 2011 by Senju and co-authors. Their CAR-iMs bearing an anti-CD20 scFv fused to FcRγ ingested and destroyed B-cell leukemic cells in vitro and in vivo [252]. In 2020, Zhang et al. generated iMs expressing CAR specific to CD19 or mesothelin. They observed that both CAR-iMs expressed typical macrophage markers. Upon activation with targeted cells, these CAR-iMs were polarized toward the pro-inflammatory M1 subtype and showed antigen-dependent anti-tumor activities in vitro and in vivo [205]. In 2020, Gutbier et al. have developed a technique to scale up the production of cells resembling tissue-resident macrophages. These iMs were capable of phagocytosis, cellular cytotoxicity, and secretion of pro-inflammatory factors and were also amenable to genetic manipulation at the macrophage-like progenitor stage [253].

The iPSCs can also be used to produce functional neutrophils. Recently, Chang et al. generated neutrophils with glioblastoma-targeting CAR from hPSCs, which displayed enhanced anti-tumor cytotoxicity both in vitro and in vivo [254].

#### Limitations

The possibility of obtaining iPSCs from nearly any somatic cells, the ease of producing iPSC in unlimited quantities, and the feasibility of genetic modification of iPSCs make them an attractive cell source for CAR technology; however, to make iPSCs clinical use a reality, ethical concerns, methodological, and regulatory issues need to be solved. The first and foremost concern regarding iPSCs is their safety, which could be addressed by precisely evaluating the biodistribution, persistence, and the possibility of tumorigenicity of cells reprogrammed from iPSCs. Also, further methodological studies are needed to increase the yield, purity, homogeneity, differentiation efficiency, and stability of iPSCs, while decreasing production costs [251].

## Conclusion and future perspective

All cells discussed in this review are predominantly used for generation CARs bearing scFv and not for VHH-based CARs, while CARs containing nanobodies have shown promising clinical outcomes; as evidence, the sixth CAR-T cell product (CARVYKTI, Janssen-Cilag International NV) recently approved by US FDA has utilized a VHH dimer as its antigen recognition domain [5]. The VHH domain, alternatively known as nanobody, is the smallest fragment with an antigen-binding capability similar to conventional antibodies in affinity and specificity [6]. Characteristics such as small size, high solubility and stability, low immunogenicity, high tissue penetration, and no need for additional folding and assembly steps or linker optimization due to the lack of variable light chain make nanobodies a promising alternative to scFvs in CARs. Furthermore, as VHHs can access epitopes that are hard or impossible to reach by scFvs, they are more favorable than scFv to be used as the antigen recognition domain of CARs, particularly for solid tumors [6]. The first report about the successful use of nanobodies in the CAR constructs emerged from our lab, where CAR-modified T cells used an anti-MUC1 VHH as the target-binding domain [255]. We have observed promising results using VHH-based CAR-T cells against solid tumor cell lines in our lab [256, 257]. As VHHs own advantages over scFvs for solid tumors [6], transferring VHH-based CAR into cells other than T cells that can penetrate deep tumors may provide an ideal immunotherapy platform against solid tumors.

## Data Availability

Not applicable.

## Abbreviations

CAR: Chimeric Antigen Receptor
FDA: Food and Drug Administration
MHC: Major Histocompatibility Complex
scFv: Single-Chain Variable Fragment
CM: Central Memory; EM, Effector Memory
B2M: β-2 Microglobulin
TCR: T Cell Receptor
GvHD: Graft-Versus-Host Disease
HLA: Human Leukocyte Antigen
ZFNs: Zinc-Finger Nucleases
TALEN: Transcription Activator-Like Effector Nucleases
CRISPR/Cas9: Clustered Regularly Interspaced Short Palindromic Repeats/ CRISPR-associated protein 9
TRAC: Constant Chain of TCRα Gene
CR: Complete Remission
PFS: Progression-Free-Survival
ADR: Alloimmune Defense Receptor
DSAs: Donor-specific Anti-HLA Antibodies
CRS: Cytokine Release Syndrome
ICU: Intensive Care Unit
Treg: Regulatory T
MAIT: Mucosal-Associated Invariant T
NKT: Natural Killer T
NK: Natural Killer
HSPCs: Hematopoietic Stem/Progenitor Cells
iPSCs: Induced Pluripotent Stem Cells
PBMCs: Peripheral Blood Mononuclear Cells
FOXP3: Forkhead Box P3
TSDR: Treg-Specific Demethylated Region
tTregs: Thymus-Derived Tregs
pTregs: Peripheral-Derived Tregs
PB: Peripheral Blood
UCB: Umbilical Cord Blood
T1D: Type 1 Diabetes
MS: Multiple Sclerosis
CNS: Central Nervous System
MOG: Myelin Oligodendrocyte Glycoprotein
CV: Citrullinated Vimentin
RA: Rheumatoid Arthritis
AILD: Autoimmune Liver Diseases
AAV: Adeno-Associated Virus
R/R: Relapsed/Refractory
ALL: Acute Lymphocytic Leukemia
LNs: Lymph Nodes
pAgs: Phosphoantigens
BTN: Butyrophilin
MIC: MHC Class I Related-Chain Antigens
IGF-1: Insulin-Like Growth Factor-1
TRAIL: TNF-Related Apoptosis-Inducing Ligand
FasL: Fas Ligand
ADCC: Antibody-Dependent Cellular Cytotoxicity
NKRs: NK Receptors
NKG2D: Natural-Killer Group 2 Member D
NCRs: Natural Cytotoxicity-Triggering Receptors
DCs: Dendritic Cells
ZOL: Zoledronate
mRCC: Metastatic Renal Cell Carcinoma
CRC: Colorectal Cancer
TNBC: Triple-Negative Breast Cancer
NKG2DL: NKG2D Ligands
CCRs: Chimeric Costimulatory Receptors
5-OERU: 5-(2-Oxoethylideneamino)-6-D-ribitylaminouracil
5-OP-RU: 5-(2-Oxopropylideneamino)-6-D-ribitylaminouracil
TAM: Tumor-Associated Macrophage; iNKT, Invariant NKT
DP: Double-Positive
α-GalCer: α-Galactosylceramide
MDSCs: Myeloid-Derived Suppressor Cells
TME: Tumor Microenvironment
cGMP: Current Good Manufacturing Practice
CSPG4: Chondroitin Sulfate Proteoglycan-4
BCMA: B Cell Maturation Antigen
CLP: Common Lymphoid Progenitor
BM: Bone Marrow
HSCT: Hematopoietic Stem Cell Transplants
hESCs: Human Embryonic Stem Cells
CMP: Common Myeloid Progenitor
ROS: Reactive Oxygen Species
RNS: Reactive Nitrogen Species
iNOS: Inducible Nitric Oxide Synthase
NO: Nitric Oxide
ECM: Extracellular Matrix
TLRs: Toll-Like Receptors
PRRs: Pattern Recognition Receptors
Megf10: Multiple EGF-like-Domains 10
FcRγ: γ Subunit of Fc Receptors
MMPs: Matrix Metalloproteinase
Ad: Adenoviral
CIR: Chimeric Immune Receptor
GVT: Graft-Versus-Tumor
CEA: Carcinoembryonic Antigen
CAR-iT: IPSC-Derived CAR-T
ATOs: Artificial Thymic Organoids
B7-H3: B7 Homolog-3 Protein
hnCD16: High-Affinity, Non-Cleavable CD16
DGK: Diacylglycerol Kinase
IL-7RF: IL-7 Receptor Fusion
iNK: IPSC-Derived NK
GPC3: Glypican 3
EGFR: Epidermal Growth Factor Receptor
GBM: Glioblastoma Multiforme
CAR-iM: IPSCs-Derived CAR-Macrophage
